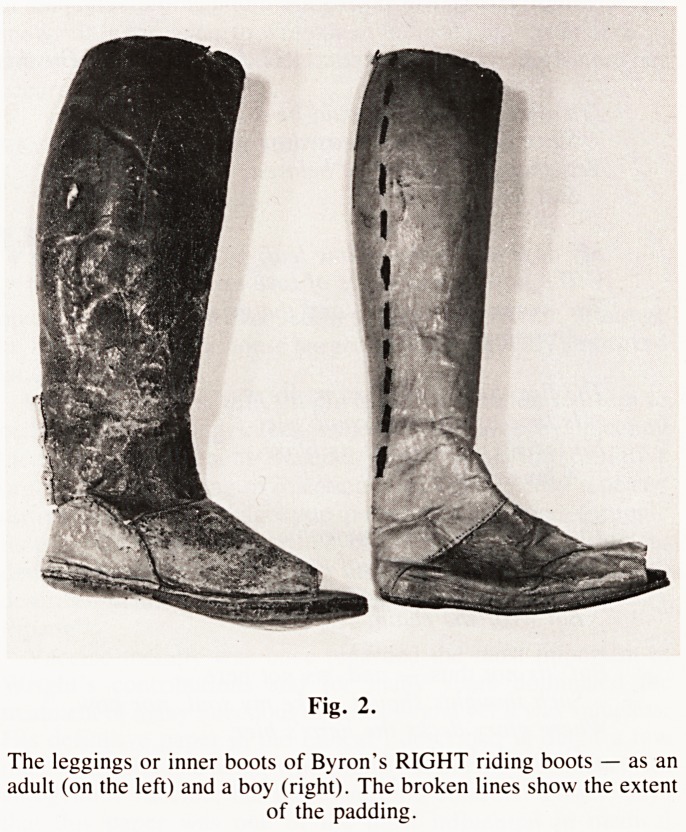# Pathos and Pathology in the Life of Lord Byron

**Published:** 1991-12

**Authors:** Roger Celestin


### Pathos and Pathology in the life of Lord Byron

**Roger Celestin, MB, BS, FRCS**

George Gordon Byron died of blackwater fever in his 37th year in the mosquito infested marshes of Missolonghi, having written with great premonition three weeks previously his last poem.

Byron had believed all his life that he would die in his 37th year and was very conscious of the family background he had inherited. His great-grandfather had been a bad tempered gentleman dubbed "the wicked Lord". His grandfather though an Admiral of some distinction had been nicknamed "Foulweather Jack" by his crew, while his own father carried the appelation of the "mad lord".

Byron was born lame and without a title in an impecunuous household. His mother kept on talking about his lameness and was insenstitive about it; even on one occasion calling her son a "lame brat". This had marked the boy and throughout his life he strove to conceal his infirmity. As a result the nature of his lameness became the subject of much discussion after his death, and much speculation surrounds it to this very day.

Moore, his biographer; Galt, a close friend on his Eastern tours and Lady Blessington another close friend could not state which foot it was. Hobhouse his everyday companion at Cambridge had no doubt it was the right foot; while Gentleman Jackson, his boxing instructor, and Teresa Guiccoli his last and longest mistress thought it was his left.

Byron had been examined by John Hunter soon after his birth, while Matthew Baillie, John Hunter's nephew, saw Byron when aged 11. Direct and indirect records of their observations incriminate the right foot — as do all the letters of Mrs. Byron on the subject.

**Fig. 1.**

The lasts on which Byron's shoes were made. They are symmetrical and of the same size.

To add to the confusion there are, in the Nottingham Museum, a pair of lasts on which Byron's shoes and boots were made. They are symmetrical and identical! (Fig. 1). The explanation for this is to be found in another one of Byron's memorabilia, namely two right boots of his in the possession of John Murray, the publisher (Fig. 2). These are in fact leggings or inner boots. The smaller one was worn by Byron when he was probably 11 or 12; and the wider one when he had reached full adulthood, probably in Cambridge. The importance of these leggings is that both are padded on the inside aspect, corresponding to the calf, and meant to conceal a thin leg. In addition the outer side of the soles was raised by some half inch either to exert a corrective effect or, more likely, to conceal a defect. In view of these "filling" devices it is not strange that the lasts were symmetrical, producing shoes that looked essentially alike and normal.

This compounds the difficulty of reaching a clear diagnosis, and whichever one is advanced it can be made to fit the boots.

**Fig. 2.**

The leggings or inner boots of Byron's RIGHT riding boots — as an adult (on the left) and a boy (right). The broken lines show the extent of the padding.

A club foot deformity remains the most popular diagnosis; but raising the outside of the sole has been held as a means of making good an equino-valgus and a shortened tendo-Achilles, deemed the commonest foot deformity seen in a mild spastic monoplegia. Dennis Browne attributes such a deformity to dysplasia or to a minor form of dysrapphism and argues that the deformity would fit well inside the boots and could be easily overcome by an intelligent patient. However, we must remember that the deformity was present at birth as confirmed by Hunter.

To complicate matters, Byron's entry into this world was not straightforward. His head, at delivery, was wrapped up in an amniotic caul which had to be pulled off. Did baby Byron suffer transient asphyxia at birth? Superstition has it that such cauls are lucky omens protecting one against drowning. Byron's caul is said to have been bought by a Mr. Hanson who presented it to his brother Captain Manson — but his ship promptly sank and he was drowned . . .

Amongst the other disorders attributed to Byron to explain his death in his 37th year, as well as those of his two previous ancestors about the same age — hence Byron's obsession — was diabetes. Thirst and lassitude were features of the last months of his life, and exercise was said to improve these. Evidence is indirect and little else supports the diagnosis.

Byron's various pathologies will remain enigmas; but had it not been for them it is doubtful whether he would have been driven to the great heights of glory and fame that he attained.

His efforts at overcoming his infirmity and genetic inheritance, combined with his deep sensitivity and great intellect, gave him a drive that few could match. He found himself in a society that was corrupt and full of hypocrisy and he paid heavily for pointing it out. He could be considered, politically, as the first "socialist" lord in the Upper House, and his remark that "God will not always be a Tory" displeased the 'Conservatives' of his day.

What he could not achieve on land he was able to do in water. He was an outstanding swimmer and, like Leander, crossed the Hellespont with ease. Dancing allowed him to hide his lame gait and he welcomed the Waltz from Germany, as he did Hock. This was part of his Hedonism, which is so well expressed in his writings. His lyrical poems are amongst the very best in English Literature, while some of his longer works are full of realism.

History has unjustly concentrated more on his weaknesses than on his talents and this is perhaps the price that someone who started life in obscurity has to pay for finding himself in the floodlights of immortality.

***22 January 1824: On this day I completed my 36th year.***

*'Tis time this heart should be unmoved,*
*Since others it has ceased to move:*
*Yet, though I cannot be beloved,*
*Still let me love!*

*My days are in the yellow leaf;*
*The flowers and fruits of love are gone;*
*The worm, the canker, and the grief*
*Are mine alone!*

*The fire that on my bosom preys*
*Is lone as some volcanic isle;*
*No torch is kindled at its blaze —*
*A funeral pile.*

*The hope, the fear, the jealous care,*
*The exalted portion of the pain*
*And power of love, I cannot share,*
*But wear the chain.*

*But 'tis not* thus *— and 'tis not* here *—*
*Such thoughts should shake my soul, nor* now,
*Where glory decks the hero's bier,*
*Or binds his brow.*

*The sword, the banner, and the field,*
*Glory and Greece, around me see!*
*The Spartan, borne upon his shield,*
*Was not more free.*

*Awake! (not Greece — she* is *awake!)*
*Awake, my spirit! Think through* whom
*Thy life-blood tracks its parent lake,*
*And then strike home!*

*Tread those reviving passions down,*
*Unworthy manhood! — unto thee*
*Indifferent should the smile or frown*
*Of beauty be.*

*If thou regrett'st thy youth,* why live?
*The land of honourable death*
*Is here: — up to the field, and give*
*Away thy breath!*

*Seek out — less often sought than found —*
*A soldier's grave, for thee the best;*
*Then look around, and choose thy ground,*
*And take they rest.*

## Figures and Tables

**Fig. 1. f1:**
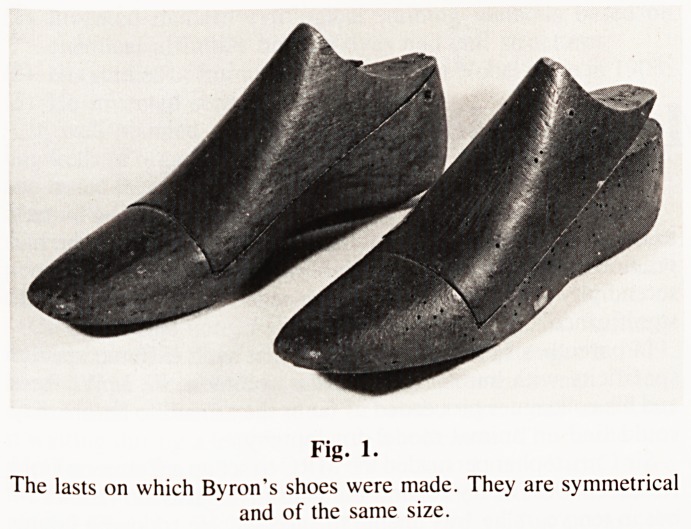


**Fig. 2. f2:**